# Robust Polarization-Domain Adaptive Anti-Jamming via Forgetting-Factor Covariance Estimation and Adaptive Diagonal Loading

**DOI:** 10.3390/s26134110

**Published:** 2026-06-29

**Authors:** Yuancong Xiong, Huafeng He, Buma Xiao, Liyuan Wang, Zhen Li

**Affiliations:** College of Missile Engineering, Rocket Force Engineering University, Xi’an 710025, China; 17829696712@163.com (Y.X.); bumaxiao@163.com (B.X.); hansw87@163.com (L.W.); 19209352359@163.com (Z.L.)

**Keywords:** adaptive beamforming, anti-jamming, covariance estimation, diagonal loading, forgetting factor, polarization-domain radar, robust MVDR

## Abstract

To address robust polarization-domain adaptive anti-jamming for dual-polarized radars with limited secondary data and time-varying interference, this paper proposes a covariance-reliability-driven MVDR framework based on forgetting-factor covariance estimation and adaptive diagonal loading. The forgetting-factor recursion assigns larger weights to recent jammer-plus-noise snapshots to track nonstationary interference, while the adaptive loading coefficient is jointly controlled by sample deficiency and covariance condition-number degradation to improve inversion stability. Unlike many robust adaptive beamforming methods that require steering-vector uncertainty sets, mismatch distributions, or subspace information, the proposed method relies only on secondary data and a small set of scalar design parameters. Simulation results based on a synthetic dual-polarized array model show that the proposed method achieves competitive output SINR, effective jammer suppression, and improved robustness to moderate DOA and polarization mismatch under limited-snapshot and time-varying interference conditions. Complexity analysis indicates that the proposed method has the same dominant computational order as standard covariance-based MVDR beamforming, apart from condition-number evaluation. The present validation is simulation-based, and further verification using measured polarimetric radar data, realistic propagation models, or hardware experiments is still required.

## 1. Introduction

Adaptive anti-jamming has long been a central topic in array and radar signal processing because practical systems often operate in electromagnetically contested environments with strong intentional interference, limited training data, and rapidly changing propagation conditions. The classical development of adaptive arrays established the main algorithmic foundations of the field, including adaptive antenna systems, the Capon/MVDR principle, linearly constrained adaptive processing, and finite-sample convergence analysis [1,2,3,4], and advanced reconfigurable array designs for radar applications [5]. These foundations remain essential in modern radar anti-jamming, but they also reveal a persistent difficulty: when the covariance matrix is estimated from limited or contaminated secondary data, the resulting adaptive beamformer can become highly sensitive to modeling errors and numerical instability.

For polarization-domain radar or navigation anti-jamming, the problem is even more challenging because dual-polarized sensing increases the available degrees of freedom while simultaneously enlarging the covariance dimension. In return, joint spatial–polarization processing can better separate the target and the jammer when angular discrimination alone is insufficient. Meanwhile, the additional polarization degree of freedom has motivated a series of space-time-polarization and dual-polarized anti-jamming studies, including adaptive space-time-polarization cancelation [6], polarization-space-time adaptive processing [7,8], and dual-polarized MVDR-type beamforming frameworks [9,10]. These studies confirm the value of polarization diversity, but they also indicate that the increased model dimension makes covariance estimation more vulnerable to limited snapshots and time-varying interference.

A common practical baseline is sample matrix inversion (SMI) within the MVDR framework. From early robust adaptive beamforming practice to later diagonal-loading analyses, it has been well recognized that direct use of the sample covariance matrix may lead to substantial performance degradation when the training support is insufficient or the presumed signal model is imperfect [9,10,11,12]. Fixed diagonal loading can partially improve numerical stability, but choosing a single loading factor that works well over different snapshot regimes and jammer conditions is difficult. These methods are conceptually related to data-dependent diagonal loading and highlight the necessity of adaptive regularization. Among these studies, the fully automatic diagonal loading method of Du et al. [13] is particularly relevant to the present work. Their method aims to determine the loading level automatically in a standard Capon beamforming framework, where a shrinkage-type enhanced covariance estimate is used to avoid manual selection of the diagonal loading parameter. This line of work is important because it demonstrates that the loading level should be data-dependent rather than fixed empirically. However, the objective of the present paper is different. Instead of designing a general-purpose parameter-free loading rule for conventional Capon beamforming, this work focuses on the covariance-reliability problem in dual-polarized joint spatial–polarization anti-jamming, where the covariance dimension is enlarged and the secondary data may be simultaneously limited and time-varying. Therefore, the proposed loading coefficient is coupled with a forgetting-factor covariance estimator and is explicitly governed by two reliability indicators, namely snapshot deficiency and condition-number degradation. This design makes the loading level responsive not only to the covariance scale, but also to the effective sample support and the numerical stability of the joint spatial–polarization covariance estimate. Furthermore, shrinkage covariance estimators provide principled, data-driven regularization alternatives that optimize the bias-variance tradeoff and are conceptually related to the adaptive loading approach proposed here [14,15]. It is also worth noting that covariance-matrix estimation is important not only in MVDR-type adaptive beamforming but also in subspace-based high-resolution methods such as MUSIC [16]. In HFSWR and related array-processing applications, MUSIC uses the covariance eigenstructure and the separation between signal and noise subspaces for high-resolution parameter estimation [17]. Its performance can also be affected by limited snapshots, coherent sources, colored noise, and covariance-estimation errors. Although this paper focuses on robust MVDR beamforming rather than MUSIC-based direction finding, this connection suggests that subspace-based covariance processing may be useful for future extensions. Likewise, when jammer power, direction, or polarization varies over time, uniformly averaging all historical samples may no longer represent the current interference environment accurately.

To address these issues, robust adaptive beamforming (RAB) has evolved along several major directions. Representative early and classical approaches include projection-based robust beamforming [18], diagonal-loading-based robustness [19,20], covariance-matrix tapering [21], and Bayesian formulations. The principles of minimum-variance robust adaptive beamforming have been systematically reviewed in [22]. Later developments include worst-case optimization [23], robust Capon beamforming and diagonal loading [24], general-rank formulations [25], doubly constrained designs [26,27], and robust minimum-variance beamforming with uncertainty sets [28]. More recent extensions have also considered eigenvalue beamforming and subspace selection [29], probabilistic robustness [30], sequential-programming solvers, and steering-vector estimation with minimal prior information [31]. Another major paradigm is robust adaptive beamforming based on interference-plus-noise covariance matrix reconstruction [32,33]. While these methods are highly effective in many scenarios, many of them require relatively strong prior information, such as steering-vector uncertainty sets, mismatch distributions, precise integration bounds for the interference angular sectors, signal subspaces, or rank knowledge.

The above observations motivate a different positioning for the present work. Instead of explicitly reconstructing the desired steering vector or imposing a prescribed mismatch set, this paper improves robustness from the covariance-estimation side. Specifically, a forgetting-factor recursion is used to emphasize recent secondary snapshots and thereby track nonstationary jammer statistics, while an adaptive diagonal loading rule is introduced to stabilize inversion when the covariance estimate becomes unreliable. The loading level is linked to two directly interpretable indicators: sample deficiency and covariance condition number. In this way, the proposed method is especially suited to the present polarization-domain anti-jamming problem, where the core challenge is not explicit steering-vector reconstruction, but reliable covariance-estimation under limited and time-varying secondary data. It should be emphasized that the individual tools employed in this work, including forgetting-factor covariance updates, diagonal loading, and MVDR beamforming, are well-established, classical techniques in adaptive filtering and array signal processing. The primary contribution of this paper does not lie in reinventing these individual components. Instead, the proposed framework is fundamentally driven by the critical issue of covariance estimation reliability unique to dual-polarized anti-jamming, where the joint spatial–polarization dimension is inherently enlarged, and the available secondary data often simultaneously suffer from sample deficiency and nonstationarity. Unlike fixed diagonal loading, whose regularization strength typically relies on manual tuning and remains invariant across different snapshot regimes, the proposed loading coefficient is adaptively and jointly governed by a sample-deficiency penalty and a condition-number penalty. Furthermore, unlike many robust adaptive beamforming schemes that rely on stringent assumptions regarding steering-vector uncertainty sets, mismatch distributions, or subspace ranks, the proposed method exclusively utilizes secondary data and scalar reliability metrics directly extracted from the estimated covariance matrix. Therefore, the proposed method should be properly envisioned as a data-driven covariance regularization framework tailored for polarization-domain anti-jamming, rather than a new mathematical variant of the MVDR beamforming. The main contributions of this paper are as follows:

1. A forgetting-factor covariance-estimation strategy is introduced for joint spatial–polarization anti-jamming. By assigning larger weights to more recent secondary snapshots, the proposed estimator can track time-varying jammer statistics more effectively than the conventional sample covariance matrix.
2. A covariance-reliability-driven adaptive diagonal loading mechanism is proposed for the joint spatial–polarization covariance matrix. Different from existing automatic diagonal loading methods for conventional Capon beamforming, the proposed loading level is coupled with the forgetting-factor covariance estimate and is jointly controlled by snapshot deficiency and condition-number degradation. Therefore, the regularization strength is explicitly linked to the reliability of the covariance estimate under limited and time-varying secondary data, without relying on steering-vector uncertainty sets, mismatch distributions, or subspace-rank information.
3. A comprehensive simulation benchmark is established, including shrinkage-based MVDR, automatic diagonal-loading MVDR, covariance-reconstruction MVDR, worst-case robust MVDR, Eigenspace-MVDR, and several ablation variants of the proposed method. Detailed simulations and complexity analysis demonstrate that the full method achieves a favorable overall tradeoff among output SINR, jammer suppression, numerical conditioning, and robustness to moderate model mismatch.

The remainder of this paper is organized as follows. Section 2 introduces the dual-polarized array signal model and formulates the covariance-estimation problem. Section 3 presents the proposed forgetting-factor adaptive diagonal loading method and analyzes its theoretical properties. Section 4 reports simulation results, including SINR performance, mismatch robustness, spatial–polarization responses, numerical stability, and ablation studies. Section 5 concludes the paper and discusses future work.

## 2. Signal Model and Problem Formulation

### 2.1. Dual-Polarized Array Model

**Figure 1:**
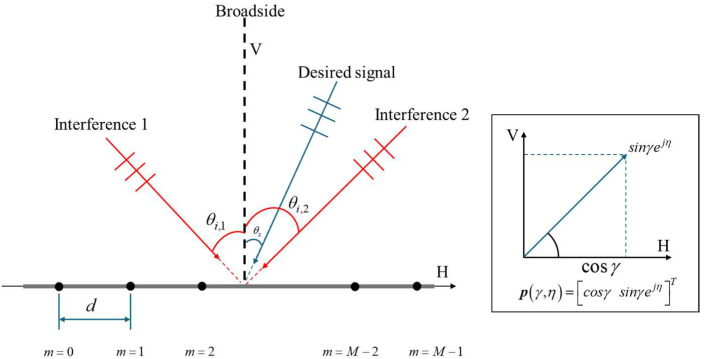
Geometry of the dual-polarized ULA and angle definitions.

Consider a dual-polarized uniform linear array (ULA) with (M) spatial sensor locations in a two-dimensional azimuth-plane geometry, as illustrated in Figure 1. The ULA is placed along the horizontal axis, and the azimuth angle θ is measured from the array broadside direction. The desired signal and the q-th jammer arrive from directions $\theta_{s}$ and $\theta_{i,q}$, respectively. Each sensor location contains two orthogonal polarization channels, so the dimension of the joint spatial–polarization snapshot is

(1) D=2M,

The polarization state is described by the auxiliary angle γ and the phase difference η. Specifically, γ controls the relative amplitude ratio between the two orthogonal polarization components, while η denotes their phase difference.

The steering-vector expression used below corresponds to this two-dimensional ULA geometry. For other array geometries, such as circular, planar, or conformal arrays, the spatial steering vector should be replaced by the corresponding array manifold determined by the actual sensor positions, element patterns, polarization bases, mutual coupling, and calibration characteristics.

For a narrowband far-field signal arriving from direction θ, the spatial steering vector is

(2) $$a_{sp}\left(\theta \right)={\left[1,e^{j2\pi \frac{d}{\lambda }sin\theta },\cdots ,e^{j2\pi \frac{d}{\lambda }\left(M-1\right)sin\theta }\right]}^{T},$$

where d is the inter-element spacing and λ is the wavelength. The polarization state is parameterized by the auxiliary angle γ and the phase difference η, with

(3) $$p\left(\gamma ,\eta \right)=\left[\begin{array}{c} cos\gamma \\ sin\gamma e^{j\eta } \end{array}\right],$$

Based on the foundational vector-sensor array processing model [34], the joint spatial–polarization steering vector is then

(4) $$a\left(\theta ,\gamma ,\eta \right)=a_{sp}\left(\theta \right)⊗p\left(\gamma ,\eta \right),$$

where ⊗ is the Kronecker product.

Assume one desired target and Q hostile jammers. The received snapshot at time index k is modeled as

(5) $$x\left(k\right)=\sqrt{P_{s}}s\left(k\right)a_{s}+\overset{Q}{\underset{q=1}{\sum }}\sqrt{P_{i,q}}i_{q}\left(k\right)a_{i,q}+n\left(k\right),$$

where $a_{s}=a\left(\theta_{s},\gamma_{s},\eta_{s}\right)$ is the target steering vector, $a_{i,q}=a\left(\theta_{i,q},\gamma_{i,q},\eta_{i,q}\right)$ is the steering vector of the q-th jammer, and $n\left(k\right)∼CN\left(0,\sigma_{n}^{2}I\right)$.

### 2.2. Secondary Data and Covariance Matrices

Adaptive anti-jamming uses secondary data that contain jammer-plus-noise components only:

(6) $$x_{sec}\left(k\right)=\overset{Q}{\underset{q=1}{\sum }}\sqrt{P_{i,q}}i_{q}\left(k\right)a_{i,q}+n\left(k\right),$$

The interference-plus-noise covariance matrix is

(7) $$R_{in}=E\left\{x_{sec}\left(k\right)x_{sec}^{H}\left(k\right)\right\}=R_{i}+R_{n},$$

where

(8) $$R_{i}=\overset{Q}{\underset{q=1}{\sum }}P_{i,q}a_{i,q}a_{i,q}^{H},$$

and

(9) $$R_{n}=\sigma_{n}^{2}I,$$

The target covariance matrix is

(10) $$R_{s}=P_{s}a_{s}a_{s}^{H},$$

Given the presumed target steering vector ${\hat{a}}_{s}$ and an estimate $\hat{R}$ of the jammer-plus-noise covariance matrix, the MVDR weight vector is

(11) $$w=\frac{{\hat{R}}^{-1}{\hat{a}}_{s}}{{\hat{a}}_{s}^{H}{\hat{R}}^{-1}{\hat{a}}_{s}},$$

The output SINR is

(12) $$SINR_{out}=\frac{w^{H}R_{s}w}{w^{H}R_{in}w},$$

To visualize anti-jamming behavior, the normalized spatial response and the normalized polarization response are defined as

(13) $$B_{\theta }\left(\theta \right)=\frac{{\left|w^{H}a\left(\theta ,\gamma ,\eta \right)\right|}^{2}}{\underset{\theta }{\max}{\left|w^{H}a\left(\theta ,\gamma ,\eta \right)\right|}^{2}},$$

and

(14) $$B_{\gamma }\left(\gamma \right)=\frac{{\left|w^{H}a\left(\theta_{s},\gamma ,\eta_{s}\right)\right|}^{2}}{max_{\gamma }{\left|w^{H}a\left(\theta_{s},\gamma ,\eta_{s}\right)\right|}^{2}},$$

In polarization-domain anti-jamming, three practical difficulties dominate the performance of the MVDR filter in (11).

1. Limited snapshots: the conventional SMI covariance estimate:

(15) $${\hat{R}}_{SMI}=\frac{1}{K}\overset{K}{\underset{k=1}{\sum }}x_{sec}\left(k\right)x_{sec}^{H}\left(k\right),$$

The estimate in (15) is formed from K secondary snapshots, where K denotes the number of jammer-plus-noise training samples and D=2M is the dimension of each joint spatial–polarization snapshot. When K is comparable to, or smaller than D, ${\hat{R}}_{\mathrm{SMI}}$ may become inaccurate or ill-conditioned.

2. Time-varying jammers: if jammer power, DOA, or polarization changes during the observation interval, uniform averaging over old and new snapshots no longer matches the current interference state well.

3. Moderate model mismatch: in practice, the presumed steering vector $a_{s}$ may deviate from the true steering vector $a_{s}$ due to DOA error, polarization error, calibration imperfection, or channel perturbation. The present study does not explicitly reconstruct $a_{s}$; instead, it seeks to improve tolerance to moderate mismatch by stabilizing the covariance estimate and the ensuing MVDR inversion.

Accordingly, the objective of this paper is to design a robust covariance estimate $\hat{R}$ such that

1. The covariance estimate tracks time-varying jammer statistics better than SMI;

2. The inversion of $\hat{R}$ remains stable in small-snapshot and strong-jamming cases;

3. The resulting beamformer maintains a high-output SINR and deep notches, with improved tolerance to moderate DOA and polarization mismatch.

## 3. Proposed Method

**Figure 2:**
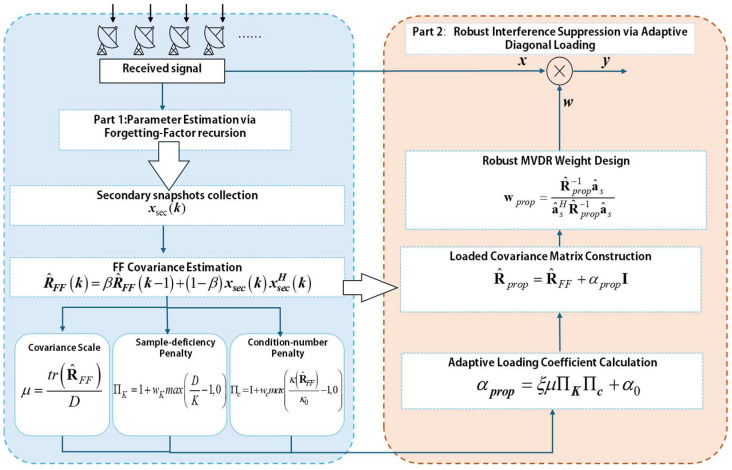
Flowchart of the proposed method.

The proposed method robustifies MVDR from the covariance-estimation side. It does not introduce a steering-vector uncertainty set, nor does it reconstruct the target steering vector from subspace information. Instead, it improves robustness through two coordinated mechanisms: forgetting-factor covariance estimation to emphasize recent jammer snapshots; adaptive diagonal loading to strengthen regularization when the covariance estimate becomes unreliable. This design is particularly suitable for the present problem, where limited and time-varying secondary data are the main bottlenecks. The method flow of this paper is shown in Figure 2.

### 3.1. Forgetting-Factor Covariance Estimation

Let $x_{sec}\left(k\right)$ be the kth jammer-plus-noise secondary snapshot. Following canonical adaptive filter theory [35], the forgetting-factor covariance recursion is

(16) $${\hat{R}}_{FF}\left(k\right)=\beta {\hat{R}}_{FF}\left(k-1\right)+\left(1-\beta \right)x_{sec}\left(k\right)x_{sec}^{H}\left(k\right),$$

where $\beta \in \left(0,1\right)$ is the forgetting factor and ${\hat{R}}_{FF}\left(0\right)=0$. Expanding the recursion gives

(17) $${\hat{R}}_{FF}\left(K\right)=\left(1-\beta \right)\overset{K}{\underset{k=1}{\sum }}\beta^{K-k}x_{sec}\left(k\right)x_{sec}^{H}\left(k\right),$$

To remove the finite-horizon bias, the normalized estimator is

(18) $${\hat{R}}_{FF}=\frac{{\hat{R}}_{FF}\left(K\right)}{1-\beta^{K}},$$

Compared with (15), (18) assigns larger weights to recent observations and is therefore better suited to nonstationary jammer environments.

Although forgetting-factor weighting improves temporal adaptability, the matrix ${\hat{R}}_{FF}$ may still be poorly conditioned. We therefore define an adaptive loading level.

The average covariance scale is

(19) $$\mu =\frac{tr\left({\hat{R}}_{FF}\right)}{D},$$

The sample-deficiency penalty is

(20) $$\Pi_{K}=1+w_{K}max\left(\frac{D}{K}-1,0\right),$$

where $w_{K}>0$. This term increases when the available snapshots are insufficient.

The condition-number penalty is

(21) $$\Pi_{c}=1+w_{c}max\left(\frac{\kappa \left({\hat{R}}_{FF}\right)}{\kappa_{0}}-1,0\right),$$

where $\kappa \left(\cdot \right)$ is the condition number, $\kappa_{0}>0$ is a reference threshold, and $w_{c}>0$ is a weighting factor.

The adaptive loading coefficient is then defined as

(22) $$\alpha_{prop}=\xi \mu \Pi_{K}\Pi_{c}+\alpha_{0},$$

where ξ>0 is the loading gain and $\alpha_{0}>0$ is a floor term. The final loaded covariance matrix becomes

(23) $${\hat{R}}_{prop}={\hat{R}}_{FF}+\alpha_{prop}I,$$

The role of each factor in (22) is clear: μ scales the loading according to the covariance energy, $\Pi_{K}$ strengthens regularization in small-snapshot cases, and $\Pi_{c}$ further increases the loading when the covariance matrix becomes excessively ill-conditioned.

This loading rule constitutes the main difference between the proposed method and conventional diagonal loading methods. A fixed loading factor applies the same regularization strength regardless of whether the covariance estimate is reliable. In contrast, the proposed loading coefficient is jointly controlled by the amount of secondary data and the spectral condition of the estimated covariance matrix. When K is sufficiently large and ${\hat{R}}_{\mathrm{FF}}$ is well-conditioned, both penalty terms are close to one, and the method behaves similarly to a lightly regularized FF-MVDR beamformer. When K is small or the covariance matrix is close to singular, the loading level automatically increases. Therefore, the proposed loading strategy is not a fixed empirical diagonal shift, but a covariance-reliability-driven regularization rule motivated by sample support and spectral conditioning.

### 3.2. MVDR Weight Based on the Proposed Covariance

Using (23), the robust MVDR problem is

(24) $$min_{w}w^{H}{\hat{R}}_{prop}w,\;\;s.t.\;w^{H}{\hat{a}}_{s}=1,$$

The corresponding Lagrangian is

(25) $$L\left(w,\lambda \right)=w^{H}{\hat{R}}_{prop}w+\lambda \left(w^{H}{\hat{a}}_{s}-1\right)+\lambda^{*}\left({\hat{a}}_{s}^{H}w-1\right),$$

Setting $\partial L/\partial w^{*}=0$ yields

(26) $${\hat{R}}_{prop}w+\lambda^{*}{\hat{a}}_{s}=0,$$

which leads to

(27) $$w_{prop}=\frac{{\hat{R}}_{prop}^{-1}{\hat{a}}_{s}}{{\hat{a}}_{s}^{H}{\hat{R}}_{prop}^{-1}{\hat{a}}_{s}},$$

The proposed method differs from a simple concatenation of classical forgetting-factor updates, diagonal loading, and MVDR beamforming; the fundamental distinction lies in how the regularization strength is determined. In traditional FF-SMI beamformers, the forgetting factor improves temporal tracking capability, but it still cannot prevent the covariance matrix from becoming ill-conditioned when the number of snapshots is small. Conversely, in traditional fixed-loading beamformers, diagonal loading improves matrix invertibility, but the loading factor typically relies on empirical selection and cannot adaptively adjust to changes in snapshot support or spectral conditioning. The proposed FF-DL method integrates these two aspects synergistically: on one hand, it utilizes the forgetting-factor covariance matrix as a temporally adaptive estimate; on the other hand, it inherently computes the loading coefficient based on the sample support and condition number of this estimate. Consequently, the regularization strength increases only when the covariance estimate becomes unreliable, rather than imposing a fixed, conservative bias.

Thus, the MVDR criterion itself is unchanged; the improvement comes from replacing the conventional covariance estimate with a more adaptive and better-conditioned one.

To avoid a long sequence of repetitive formulas, the compared MVDR-based methods are described in a compact form. In the main performance comparison, six practical methods are considered, including Shrinkage-MVDR, Auto-DL-MVDR, Recon-MVDR, Worst Case-MVDR, Eigenspace-MVDR, and the proposed FF-DL method. The ideal-covariance MVDR result is also provided as an upper performance bound. These methods differ mainly in how they handle covariance-estimation errors, covariance regularization, steering-vector mismatch, and matrix ill-conditioning.

The proposed FF-DL method combines forgetting-factor covariance tracking with reliability-driven diagonal loading. The loading coefficient is adaptively controlled by both snapshot deficiency and covariance conditioning. Therefore, the proposed method improves MVDR robustness through covariance adaptation and regularization, without requiring a separate steering-vector reconstruction stage or an explicit mismatch uncertainty bound.

To further verify the contribution of each component, several ablated variants are considered in the ablation study. FF-FDL uses the forgetting-factor covariance estimate with a fixed diagonal loading level. FF-ADL-S retains only the snapshot-deficiency-based loading term, whereas FF-ADL-C retains only the condition-number-based loading term. FF-ADL-Add adopts an additive combination of the two reliability terms. The complete FF-DL method jointly exploits forgetting-factor covariance tracking, snapshot-deficiency awareness, and condition-number-based covariance reliability.

### 3.3. Algorithm Summary and Computational Complexity Analysis

The procedure is summarized in Table 1.

Regarding the computational complexity, let D denote the joint spatial–polarization dimension, K the number of secondary snapshots, G the number of grid points used for covariance reconstruction, and $I_{\mathrm{wc}}$ the number of iterations required in the uncertainty-parameter search of Worst Case-MVDR. For covariance-based MVDR beamforming, the dominant costs are covariance estimation, matrix inversion or eigenvalue decomposition, grid-based reconstruction, and iterative robust optimization.

The complexities of the main compared methods are summarized in Table 2. Shrinkage-MVDR and Auto-DL-MVDR mainly require covariance construction and one matrix inversion. Recon-MVDR introduces an additional grid-based covariance reconstruction term. Worst Case-MVDR requires an iterative uncertainty-parameter search, while Eigenspace-MVDR involves an additional eigenvalue decomposition for subspace projection. For the proposed FF-DL method, the forgetting-factor covariance update requires $O(KD^{2})$, and the conditioning evaluation can be implemented by eigenvalue decomposition or singular value decomposition with complexity $O(D^{3})$. The snapshot-deficiency factor and the loading coefficient are scalar-level calculations.

Here, G denotes the number of reconstruction grid points, and $I_{\mathrm{wc}}$ denotes the number of iterations in Worst Case-MVDR. The ideal-covariance MVDR result is used only as an oracle upper bound and is not included as a practical method.

Although the condition-number term $\Pi_{c}$ is defined using the exact condition number of the forgetting-factor covariance matrix, it is not necessary to compute a full eigenvalue decomposition or singular value decomposition at every covariance update in practical real-time implementation. Several low-complexity strategies can be adopted. First, $\Pi_{c}$ can be updated periodically over several covariance-update intervals, while the loading coefficient is kept unchanged within adjacent intervals. Second, approximate condition-number estimates can be obtained from Cholesky or LDL decompositions, thereby avoiding a full singular value decomposition. Third, iterative methods, such as power iteration, inverse iteration, or Lanczos-based methods, can be used to estimate the largest and smallest eigenvalues efficiently. In a recursive covariance-update framework, eigenvalue estimates from the previous time instant can also be used as warm starts to reduce the number of iterations. In addition, a threshold-triggered update strategy can be adopted, where $\Pi_{c}$ is recomputed only when the covariance power level, $\Pi_{K}$, or the covariance matrix variation exceeds a predefined threshold. Therefore, the exact condition-number expression is retained in this paper to clearly describe the reliability-dependent regularization mechanism, while periodic or approximate condition-number estimation can be used in real-time radar implementation to balance robustness and computational efficiency.

### 3.4. Theoretical Properties and Performance Explanations

The proposed method improves the MVDR beamformer from the covariance-estimation side. Its robustness is not only related to the positive definiteness introduced by diagonal loading, but also to the combined effects of covariance perturbation suppression, temporal tracking, steering-vector mismatch tolerance, and covariance-uncertainty control. To make these mechanisms explicit, this section analyzes the proposed method through five aspects: the basic regularization effect of diagonal loading, the SINR-loss bound under covariance perturbation, the tracking behavior of the forgetting-factor covariance estimate, the mismatch-tolerance mechanism, and the robust-optimization interpretation of the adaptive loading rule.

#### 3.4.1. Basic Regularization Effect of Diagonal Loading

The loaded covariance matrix used by the proposed method is

(28) $${\hat{R}}_{\mathrm{prop}}={\hat{R}}_{\mathrm{FF}}+\alpha_{\mathrm{prop}}I,\;\alpha_{\mathrm{prop}}>0.$$

Since the forgetting-factor covariance estimate ${\hat{R}}_{\mathrm{FF}}$ is Hermitian positive semidefinite, for any nonzero vector Z:

(29) $$z^{H}{\hat{R}}_{\mathrm{prop}}z=z^{H}{\hat{R}}_{\mathrm{FF}}z+\alpha_{\mathrm{prop}}z^{H}z>0.$$

Thus, ${\hat{R}}_{\mathrm{prop}}$ is Hermitian positive definite and invertible. This property guarantees that the MVDR weight vector can be stably computed even when the sample covariance estimate is rank-deficient or ill-conditioned.

Let $\lambda_{\max}$ and $\lambda_{\min}$ denote the maximum and minimum eigenvalues of ${\hat{R}}_{\mathrm{FF}}$, respectively. After diagonal loading, the condition number becomes

(30) $$\kappa \left({\hat{R}}_{\mathrm{FF}}+\alpha_{\mathrm{prop}}I\right)=\frac{\lambda_{\max}+\alpha_{\mathrm{prop}}}{\lambda_{\min}+\alpha_{\mathrm{prop}}}\leq \frac{\lambda_{\max}}{\lambda_{\min}}=\kappa \left({\hat{R}}_{\mathrm{FF}}\right),$$

provided that $\lambda_{\max}\geq \lambda_{\min}>0$. Therefore, diagonal loading increases the smallest eigenvalue and reduces the condition number of the covariance matrix. These basic regularization properties provide numerical stability, but they do not by themselves fully explain the observed SINR improvement and mismatch tolerance. The following subsections further clarify these performance mechanisms.

#### 3.4.2. SINR-Loss Bound Under Covariance Perturbation

Let R denote the true interference-plus-noise covariance matrix. Under the assumption of a perfectly known steering vector, the oracle MVDR weight vector based on R is

(31) $$w_{o}=\frac{R^{-1}a_{s}}{a_{s}^{H}R^{-1}a_{s}}$$

and the corresponding output interference-plus-noise power is

(32) $$P_{o}=w_{o}^{H}Rw_{o}=\frac{1}{a_{s}^{H}R^{-1}a_{s}}$$

Define the covariance perturbation between the proposed loaded covariance matrix and the true covariance matrix as

(33) $$\Delta ={\hat{R}}_{\mathrm{prop}}-R,∥\Delta {∥}_{2}\leq \varepsilon ,$$

where ε is an upper bound on the covariance mismatch. Let $\hat{w}$ be the MVDR weight vector obtained from ${\hat{R}}_{\mathrm{prop}}$. Since $\hat{w}$ minimizes $w^{H}{\hat{R}}_{\mathrm{prop}}w$ under the constraint $w^{H}a_{s}=1$, and $w_{o}$ is also feasible for the same constraint, the following inequality holds:

(34) $${\hat{w}}^{H}{\hat{R}}_{\mathrm{prop}}\hat{w}\leq w_{o}^{H}{\hat{R}}_{\mathrm{prop}}w_{o}\leq w_{o}^{H}Rw_{o}+\varepsilon ∥w_{o}{∥}_{2}^{2}=P_{o}+\varepsilon ∥w_{o}{∥}_{2}^{2}$$

Moreover,

(35) $${\hat{w}}^{H}{\hat{R}}_{\mathrm{prop}}\hat{w}\geq \lambda_{\min}\left({\hat{R}}_{\mathrm{prop}}\right)∥\hat{w}{∥}_{2}^{2}$$

which gives

(36) $$∥\hat{w}{∥}_{2}^{2}\leq \frac{P_{o}+\varepsilon ∥w_{o}{∥}_{2}^{2}}{\lambda_{\min}\left({\hat{R}}_{\mathrm{prop}}\right)}$$

The actual output interference-plus-noise power of $\hat{w}$ is

(37) $$\hat{P}={\hat{w}}^{H}R\hat{w}$$

Using (35)–(37), a conservative upper bound can be obtained as

(38) $$\hat{P}\leq {\hat{w}}^{H}{\hat{R}}_{\mathrm{prop}}\hat{w}+\varepsilon ∥\hat{w}{∥}_{2}^{2}\leq \left(P_{o}+\varepsilon ∥w_{o}{∥}_{2}^{2}\right)\left(1+\frac{\varepsilon }{\lambda_{\min}\left({\hat{R}}_{\mathrm{prop}}\right)}\right)$$

When the desired steering vector is perfectly known, the target response under the distortionless constraint is the same for both filters. Therefore, the SINR loss relative to the oracle MVDR can be expressed as

(39) $$\Lambda_{\mathrm{SIN}R}=\frac{{\mathrm{SIN}R}_{o}}{{\mathrm{SIN}R}_{\hat{w}}}=\frac{\hat{P}}{P_{o}}$$

Combining (38) and (39) yields

(40) $$\Lambda_{\mathrm{SIN}R}\leq \left(1+\frac{\varepsilon ∥w_{o}{∥}_{2}^{2}}{P_{o}}\right)\left(1+\frac{\varepsilon }{\lambda_{\min}\left({\hat{R}}_{\mathrm{prop}}\right)}\right)$$

Equation (40) shows that the SINR loss is related to both the covariance perturbation level ε and the minimum eigenvalue of the loaded covariance matrix. Diagonal loading increases $\lambda_{\min}\left({\hat{R}}_{\mathrm{prop}}\right)$ and thereby reduces the amplification of covariance perturbations during matrix inversion. This explains why proper loading improves output SINR in small-snapshot and ill-conditioned cases. However, Equation (40) does not imply that a larger loading factor always improves performance. Increasing $\alpha_{\mathrm{prop}}$ can improve numerical stability, but excessive loading may increase the deviation from the true covariance matrix and weaken adaptive null formation. Therefore, the loading level must balance covariance stability and interference suppression capability.

#### 3.4.3. Tracking Behavior of Forgetting-Factor Covariance Estimation

Let $R\left(k\right)$ denote the true interference-plus-noise covariance matrix at time index k. Ignoring random snapshot fluctuations, the expectation form of the forgetting-factor covariance recursion can be written as

(41) $${\hat{R}}_{\mathrm{FF}}\left(k\right)=\beta {\hat{R}}_{\mathrm{FF}}\left(k-1\right)+\left(1-\beta \right)R\left(k\right)$$

Define the tracking error as

(42) $$E_{t}\left(k\right)={\hat{R}}_{\mathrm{FF}}\left(k\right)-R\left(k\right)$$

If the covariance variation rate is bounded by

(43) $$∥R\left(k\right)-R\left(k-1\right){∥}_{2}\leq \nu$$

where ν characterizes the changing speed of the jammer statistics, then

(44) $$E_{t}\left(k\right)=\beta E_{t}\left(k-1\right)+\beta \left[R\left(k-1\right)-R\left(k\right)\right]$$

Taking the spectral norm on both sides gives

(45) $$∥E_{t}\left(k\right){∥}_{2}\leq \beta ∥E_{t}\left(k-1\right){∥}_{2}+\beta \nu$$

By recursion,

(46) $$∥E_{t}\left(k\right){∥}_{2}\leq \beta^{k}∥E_{t}\left(0\right){∥}_{2}+\frac{\beta \left(1-\beta^{k}\right)}{1-\beta }\nu$$

(47) $$\underset{k\to \infty }{\lim\;\sup}∥E_{t}\left(k\right){∥}_{2}\leq \frac{\beta }{1-\beta }\nu$$

This result shows that the forgetting factor β controls the tradeoff between estimation variance and tracking bias. A larger β preserves more historical samples and reduces random fluctuation, but it also increases the tracking lag under rapidly varying interference. A smaller β responds faster to jammer variation, but the effective sample length becomes shorter and the covariance estimate may become more variable. The effective memory length of exponential forgetting can be approximated as

(48) $$L_{\mathrm{eff}}\approx \frac{1}{1-\beta }$$

For example, β=0.96 corresponds to an effective memory length of approximately 25 snapshots. Therefore, β should be selected according to the jammer variation rate and the acceptable covariance-estimation variance.

#### 3.4.4. Mismatch-Tolerance Explanation Under Steering-Vector Perturbation

The proposed method does not explicitly correct the target steering vector. Nevertheless, covariance regularization can improve tolerance to moderate DOA and polarization mismatch by reducing the sensitivity of the MVDR weight vector. Let the true target steering vector be

(49) $$a_{s}={\hat{a}}_{s}+\delta a$$

where ${\hat{a}}_{s}$ is the presumed steering vector and δa denotes the steering-vector error caused by DOA error, polarization-parameter error, array calibration error, or channel perturbation. The MVDR weight vector designed with ${\hat{a}}_{s}$ satisfies

(50) $$w^{H}{\hat{a}}_{s}=1$$

Its response to the true steering vector is

(51) $$w^{H}a_{s}=w^{H}{\hat{a}}_{s}+w^{H}\delta a=1+w^{H}\delta a$$

By the Cauchy–Schwarz inequality

(52) $$\left|w^{H}a_{s}\right|\geq 1-∥w{∥}_{2}∥\delta a{∥}_{2}$$

Therefore, a conservative sufficient condition for avoiding severe target-response collapse is

(53) $$∥\delta a{∥}_{2}<\frac{1}{∥w{∥}_{2}}$$

For the proposed loaded covariance matrix, the MVDR weight vector is

(54) $$\hat{w}=\frac{{\hat{R}}_{\mathrm{prop}}^{-1}{\hat{a}}_{s}}{{\hat{a}}_{s}^{H}{\hat{R}}_{\mathrm{prop}}^{-1}{\hat{a}}_{s}}$$

Using the eigenvalue bounds of ${\hat{R}}_{\mathrm{prop}}^{-1}$, its norm can be conservatively bounded by

(55) $$∥\delta a{∥}_{2}<\frac{∥{\hat{a}}_{s}{∥}_{2}}{\kappa \left({\hat{R}}_{\mathrm{prop}}\right)}$$

Since

(56) $$\kappa \left({\hat{R}}_{\mathrm{prop}}\right)=\frac{\lambda_{\max}\left({\hat{R}}_{\mathrm{FF}}\right)+\alpha_{\mathrm{prop}}}{\lambda_{\min}\left({\hat{R}}_{\mathrm{FF}}\right)+\alpha_{\mathrm{prop}}}$$

proper diagonal loading reduces the condition number and increases the conservative mismatch-tolerance radius in (55). This explains why the proposed method can improve tolerance to moderate DOA and polarization mismatch even without explicit steering-vector reconstruction. When the DOA and polarization errors are small, the steering-vector perturbation can be approximated by the first-order expansion

(57) $$\delta a\approx J_{\theta }\Delta \theta +J_{\gamma }\Delta \gamma +J_{\eta }\Delta \eta$$

where $J_{\theta }$, $J_{\gamma }$, and $J_{\eta }$ denote the Jacobian matrices of the joint spatial–polarization steering vector with respect to DOA, polarization auxiliary angle, and polarization phase difference, respectively. Accordingly, a conservative sufficient condition can be written as

(58) $$∥J_{\theta }{∥}_{2}\left|\Delta \theta \right|+∥J_{\gamma }{∥}_{2}\left|\Delta \gamma \right|+∥J_{\eta }{∥}_{2}\left|\Delta \eta \right|<\frac{∥{\hat{a}}_{s}{∥}_{2}}{\kappa \left({\hat{R}}_{\mathrm{prop}}\right)}$$

Equation (58) links the tolerable DOA and polarization errors to the condition number of the loaded covariance matrix. It should be emphasized that this result does not mean that covariance loading corrects steering-vector mismatch. Instead, the loading reduces the norm of the MVDR weight vector and makes the filter less sensitive to moderate mismatch.

#### 3.4.5. Robust Covariance-Uncertainty Interpretation of Adaptive Loading

The adaptive loading rule in (22) can be interpreted from a robust covariance-uncertainty viewpoint. Suppose the true covariance matrix lies in the following spectral-norm uncertainty set:

(59) $$U\left(\rho \right)=\left\{R:R={\hat{R}}_{\mathrm{FF}}+\Delta ,\Delta =\Delta^{H},∥\Delta {∥}_{2}\leq \rho \right\}$$

where ρ is the covariance-uncertainty radius. The corresponding Worst Case-MVDR problem is

(60) $$\underset{w}{\min}\underset{R\in U\left(\rho \right)}{\max}w^{H}Rw,\;s.t.w^{H}a_{s}=1$$

For any fixed weight vector w

(61) $$\underset{∥\Delta {∥}_{2}\leq \rho }{\max}w^{H}\left({\hat{R}}_{\mathrm{FF}}+\Delta \right)w=w^{H}{\hat{R}}_{\mathrm{FF}}w+\rho ∥w{∥}_{2}^{2}$$

Therefore, the worst-case problem in (60) is equivalent to the diagonally loaded MVDR problem in (62).

(62) $$\underset{w}{\min\;}w^{H}\left({\hat{R}}_{\mathrm{FF}}+\rho I\right)w,\;s.t.w^{H}a_{s}=1$$

This equivalence is consistent with the classical interpretation of diagonal loading as a robust MVDR/Capon design under covariance-estimation errors or norm-bounded uncertainty models [20,24,28].

This equivalence shows that diagonal loading can be regarded as the robust MVDR solution under a spectral-norm-bounded covariance uncertainty set, where the loading coefficient corresponds to the uncertainty radius. Based on this interpretation, the proposed method approximates the uncertainty radius as

(63) $$\rho \approx \alpha_{\mathrm{prop}}=\xi \mu \Pi_{K}\Pi_{c}+\alpha_{0}.$$

Here, μ scales the loading according to the covariance energy, $\Pi_{K}$ characterizes the covariance uncertainty caused by limited snapshots, and $\Pi_{c}$ characterizes the inversion sensitivity caused by poor conditioning. Since the MVDR weight vector directly depends on covariance inversion, finite-sample error and inversion sensitivity jointly influence the final beamformer. Therefore, the product $\Pi_{K}\Pi_{c}$ is used to describe their coupled effect.

It should be noted that (22) is not claimed to be the unique globally optimal loading level under an exact statistical model. Rather, it is a closed-form approximation of the covariance uncertainty radius driven by sample deficiency and matrix conditioning. This interpretation connects the proposed adaptive loading rule with robust MVDR design and explains why the loading level automatically increases when the covariance estimate becomes less reliable.

#### 3.4.6. Summary of Theoretical Implications

In summary, the robustness of the proposed method can be understood from three aspects. First, the forgetting-factor recursion enables the covariance estimate to track time-varying jammer statistics at a rate controlled by β. Second, adaptive diagonal loading increases $\lambda_{\min}\left({\hat{R}}_{\mathrm{prop}}\right)$ and reduces $\kappa \left({\hat{R}}_{\mathrm{prop}}\right)$, thereby suppressing the amplification of covariance perturbations during MVDR inversion and reducing the SINR-loss bound. Third, the improved conditioning reduces the MVDR weight-vector norm and enhances tolerance to moderate DOA and polarization mismatch. Therefore, the robustness of the proposed method is not only due to the positive definiteness of diagonal loading, but also due to the joint effects of temporal tracking, covariance-uncertainty control, and mismatch-sensitivity reduction.

## 4. Simulation Results

To evaluate the proposed method more comprehensively, this subsection compares FF-DL with representative robust adaptive beamforming methods beyond classical SMI-type baselines. The compared methods include interference covariance matrix reconstruction methods, shrinkage covariance-estimation methods, worst-case uncertainty-set beamforming methods, and eigenspace-based robust beamforming methods. All methods adopt the same dual-polarized array model and the same joint spatial–polarization steering vector. Unless otherwise specified, the target, interference, SNR, INR, and snapshot settings are consistent with Table 3. For methods that require prior angular ranges or mismatch bounds, the assumed target direction and mismatch range used in the robustness experiments are adopted to make the comparison as fair as possible.

### 4.1. Simulation Setup and Compared Methods

The simulation follows a dual-polarized array with M=10 spatial channels, resulting in D=20 joint channels. The inter-element spacing is d=0.5λ. Unless otherwise stated, the desired target is located at $\theta_{s}=10^{{}^{\circ}}$, with a polarization auxiliary angle $\gamma_{s}=25^{{}^{\circ}}$ and phase difference $\eta_{s}=0^{{}^{\circ}}$. Two hostile jammers are placed at −30°and 40°, respectively. Their nominal polarization auxiliary angles are 70° and $35^{{}^{\circ}}$, and their nominal phase differences are 60° and −80°. The noise variance is fixed at $\sigma_{n}^{2}=1$.

A total of six practical MVDR-based beamforming methods were selected for comparison and validation in the above scenarios. The optimal benchmark obtained using the ideal covariance information is also provided as an upper performance bound.

(a) Shrinkage-MVDR method [15]: The method improves the conditioning of the sample covariance matrix by shrinking it toward a structured target matrix.

(b) Auto-DL-MVDR method [13]: The method determines the diagonal loading level automatically from the received data.

(c) Recon-MVDR method [33]: The method reconstructs the interference-plus-noise covariance matrix to improve beamforming robustness.

(d) Worst Case-MVDR method [28]: The method uses a steering-vector uncertainty set to enhance robustness against model mismatch.

(e) Eigenspace-MVDR method [29]: The method exploits subspace information to reduce covariance-estimation errors.

(f) Proposed FF-DL method. The method combines forgetting-factor covariance tracking with reliability-driven diagonal loading. The loading coefficient is adaptively adjusted according to snapshot deficiency and covariance conditioning.

All methods are tested under the same simulation conditions for a fair comparison.

The wavelength is normalized to λ=1 in the simulations, and the inter-element spacing d=0.5λ corresponds to a standard half-wavelength ULA configuration. Therefore, all array distances are expressed in wavelength units.

For the proposed method, the forgetting factor is set to β=0.96. The adaptive-loading parameters are chosen as

(64) $$\begin{array}{l} \xi =0.03,w_{K}=1.2,w_{c}=0.6\\ \kappa_{0}=500,\alpha_{0}=10^{-3}\mu \end{array}$$

These settings provide a moderate loading level at sufficient snapshots and automatically increase the loading in poorly conditioned cases.

For a fair comparison, the fixed-loading baselines use the same covariance scale:

(65) $$\alpha_{DL}=0.03\mu ,\alpha_{FDL}=0.03\mu$$

All methods use the same presumed target steering vector unless a mismatch experiment is explicitly conducted.

### 4.2. Example 1: SINR Performance in Dynamic Environments

In practical radar operations, jammer statistics are often nonstationary. To test temporal adaptability, jammer parameters are allowed to vary smoothly across the secondary data window according to the following models:

(66) $$INR_{q}\left(k\right)=INR_{q}^{\left(0\right)}+\Delta INR_{q}\sin\left(\frac{2\pi k}{K}+\phi_{q}\right)$$

(67) $$\theta_{q}\left(k\right)=\theta_{q}^{\left(0\right)}+\Delta \theta_{q}\sin\left(\frac{2\pi k}{K}+\varphi_{q}\right)$$

(68) $$\gamma_{i,q}\left(k\right)=\gamma_{i,q}^{\left(0\right)}+\Delta \gamma_{q}\sin\left(\frac{2\pi k}{K}+\psi_{q}\right)$$

where $\Delta INR_{q}=2\;\mathrm{dB}$ $\Delta \theta_{q}=2^{{}^{\circ}}$, and $\Delta \gamma_{q}=5^{{}^{\circ}}$. The phases $\phi_{q}$, $\varphi_{q}$, and $\psi_{q}$ are fixed but different for the two jammers.

**Figure 3:**
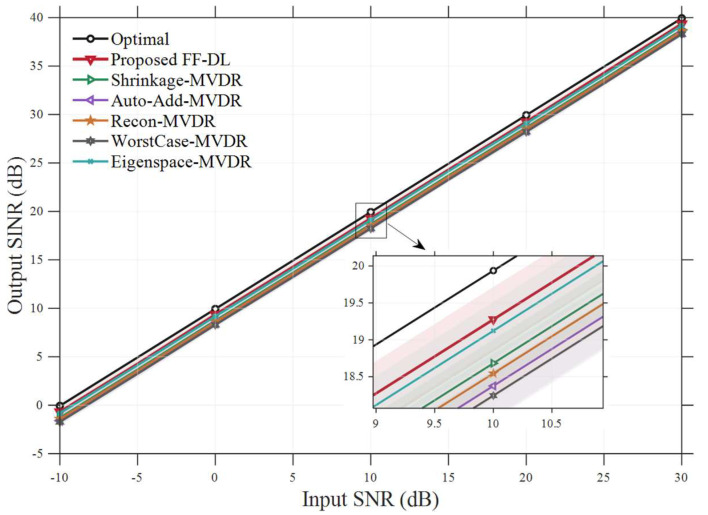
Output SINR as a function of input SNR.

Figure 3 shows the output SINR as a function of the input SNR. As expected, the output SINR of all methods increases almost linearly with the input SNR, following the same trend as the optimal benchmark. Among the practical methods, the proposed FF-DL method consistently achieves the closest performance to the optimal solution over the whole SNR range. Compared with Shrinkage-MVDR and Auto-DL-MVDR [13], the proposed method provides a higher output SINR because its loading factor is not determined only by a fixed shrinkage rule or by an automatic loading rule. Instead, it is jointly controlled by the snapshot-deficiency term and the condition-number-related reliability term. Recon-MVDR, Worst Case-MVDR, and Eigenspace-MVDR can also improve robustness to different degrees, but their covariance regularization or uncertainty modeling does not explicitly reflect the reliability of the high-dimensional joint spatial–polarization covariance estimate. Therefore, they exhibit a larger gap from the optimal benchmark. This result indicates that the proposed reliability-driven diagonal loading rule can effectively mitigate covariance-estimation error while retaining sufficient adaptive degrees of freedom.

**Figure 4:**
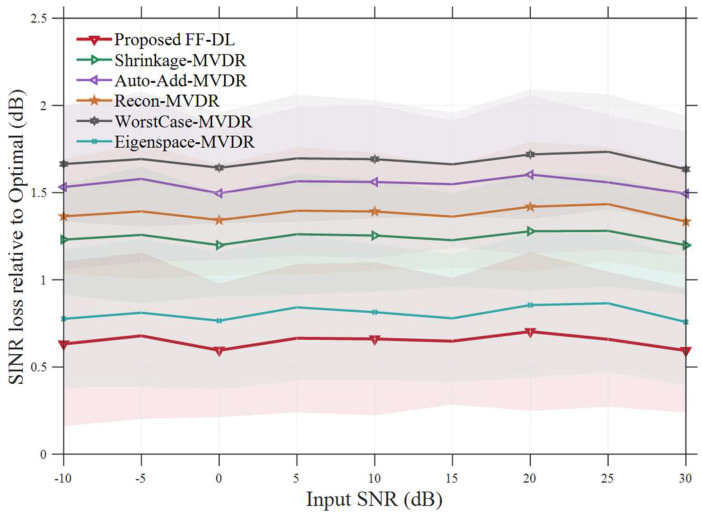
Output SINR loss relative to the optimal benchmark.

Figure 4 further plots the output SINR loss of each method with respect to the optimal benchmark. The loss of the proposed FF–DL method is the lowest among all practical methods and remains approximately within 0.6–0.7 dB across the tested SNR range. In comparison, all baseline methods exhibit larger losses. Among the compared baselines, Eigenspace–MVDR performs relatively close to the proposed method, but it still shows a larger loss because the subspace-based processing may not fully preserve the useful covariance structure in the joint spatial–polarization domain. Shrinkage–MVDR and Auto-DL–MVDR [13] improve the numerical stability of covariance inversion, but their loading or shrinkage strategies are not jointly adapted to snapshot deficiency and covariance ill-conditioning. Recon–MVDR and Worst Case–MVDR provide robustness through covariance reconstruction or uncertainty modeling, but their performance remains less competitive in the considered anti-jamming scenario. This result confirms that automatic diagonal loading alone is not sufficient to achieve the best performance in the joint spatial–polarization anti-jamming problem. The advantage of the proposed FF-DL method comes from the joint use of forgetting-factor covariance tracking and reliability-driven loading control.

**Figure 5:**
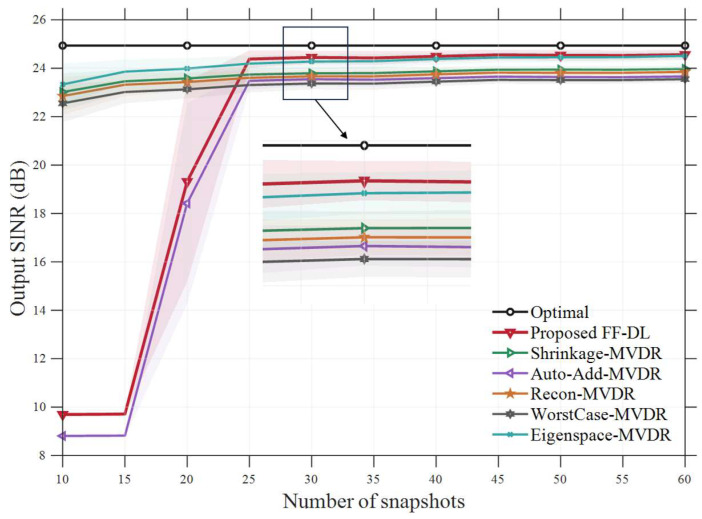
Output SINR versus number of snapshots.

Figure 5 compares the output SINR as a function of the number of snapshots. Since the joint spatial–polarization covariance matrix has an enlarged dimension, the cases with very few snapshots, such as K=10 and K=15, correspond to a severely underdetermined covariance-estimation condition. In this region, the proposed FF–DL method applies strong regularization and therefore shows a conservative output SINR. This behavior not only avoids unstable covariance inversion but also limits the adaptive interference-suppression gain when the available sample support is far below the covariance dimension.

When the number of snapshots becomes comparable to or larger than the joint covariance dimension, the proposed method rapidly recovers its adaptive capability. In particular, for K≥25, the proposed FF-DL method consistently achieves the highest output SINR among all practical methods and approaches the optimal benchmark. This demonstrates that the proposed reliability-driven loading mechanism is especially effective in the moderately limited-snapshot region, where the covariance matrix is estimable but still affected by sample deficiency and ill-conditioning.

### 4.3. Example 2: Robustness Against Model Mismatch

To evaluate tolerance to moderate model mismatch, the presumed target direction is set as ${\hat{\theta }}_{s}=\theta_{s}+\Delta \theta$ where $\Delta \theta \in \left[0,6^{{}^{\circ}}\right]$, and the presumed target polarization auxiliary angle is ${\hat{\gamma }}_{s}=\gamma_{s}+\Delta \gamma$ where $\Delta \gamma \in \left[0,15^{{}^{\circ}}\right]$.

**Figure 6:**
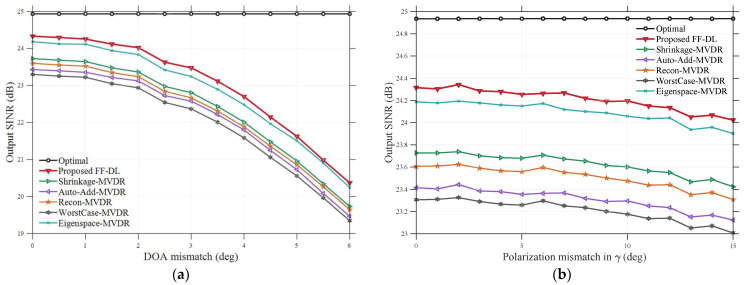
(**a**) Output SINR as a function of DOA mismatch. (**b**) Output SINR as a function of polarization mismatch.

Figure 6a evaluates the robustness of different methods against DOA mismatch. As the DOA mismatch increases, the output SINR of all methods decreases because the presumed steering vector gradually deviates from the actual target steering vector. Nevertheless, the proposed FF-DL method maintains the highest output SINR over the whole mismatch range. This indicates that the proposed covariance-reliability-driven loading rule can reduce the sensitivity of the MVDR solution to steering-vector errors. Compared with Auto-DL-MVDR and other robust MVDR variants, the proposed method provides better robustness because the loading level is jointly controlled by snapshot deficiency and covariance conditioning rather than by a fixed or purely automatic loading rule.

Figure 6b shows the output SINR under polarization mismatch in γ. Compared with the DOA mismatch case, the performance degradation caused by polarization mismatch is much milder. The proposed FF-DL method remains the best-performing method and maintains a stable SINR level over the whole tested range. This result suggests that the proposed joint spatial–polarization processing scheme can tolerate moderate polarization-parameter errors while maintaining effective jammer suppression.

### 4.4. Example 3: Spatial and Polarization Responses

After demonstrating the effectiveness of the covariance-estimation algorithm proposed in this work, we further evaluate the interference suppression capability of the proposed method. The joint spatial–polarization domain response reflects the array sensitivity of beamforming weight vectors to signals with different directional and polarization parameters. The spatial response of the proposed FF-DL method is compared with the benchmark methods, including Shrinkage-MVDR, Auto-DL-MVDR, Recon-MVDR, Worst Case-MVDR, and Eigenspace-MVDR.

**Figure 7:**
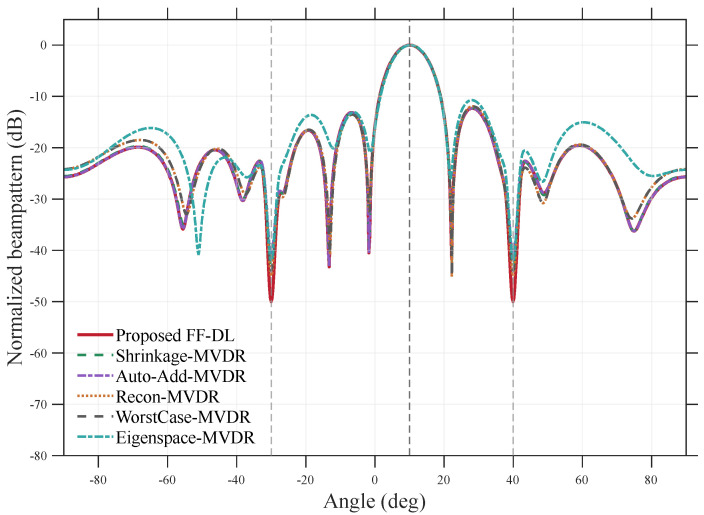
Normalized spatial beampatterns of different methods.

Figure 7 presents the normalized spatial beampatterns of the compared methods. All methods preserve the main response in the desired look direction and form spatial nulls around the jammer directions. However, the proposed FF-DL method produces deeper nulls at the dominant interference directions while maintaining a well-controlled sidelobe level. This confirms that the proposed covariance regularization does not simply increase the diagonal loading strength, but instead preserves the adaptive spatial–polarization degrees of freedom required for interference cancelation.

In contrast, some benchmark methods exhibit shallower nulls or higher sidelobe responses in certain angular regions. This explains their larger SINR loss observed in the previous figures. The beampattern result therefore provides a spatial-domain interpretation of the SINR advantage of the proposed method.

**Figure 8:**
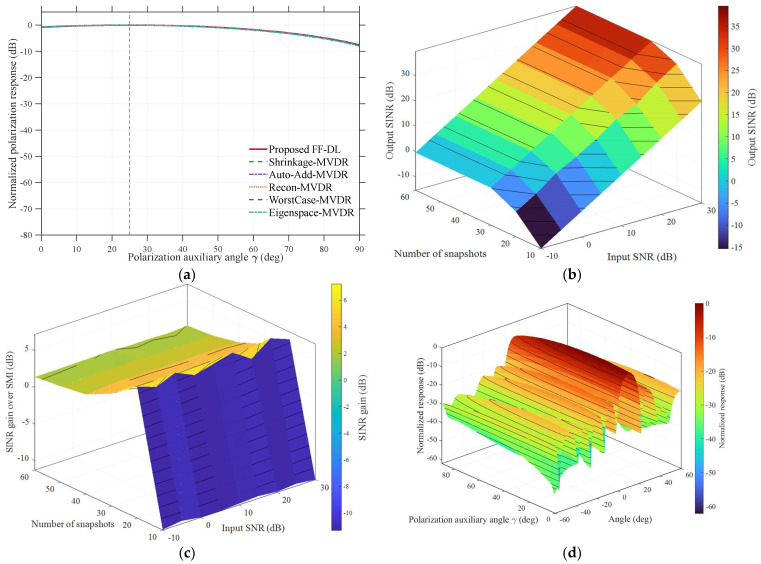
Spatial and polarization responses. (**a**) Polarization response at the target direction; (**b**) three-dimensional output SINR surface of the proposed method; (**c**) three-dimensional SINR gain surface of the proposed method over SMI; (**d**) joint spatial–polarization response of the proposed method.

Figure 8a shows the normalized polarization responses of the compared methods. The curves of different methods overlap heavily, indicating that all methods preserve a similar polarization response around the desired signal component. Therefore, the performance differences observed in the output SINR are mainly caused by the different covariance estimation and regularization strategies, rather than by severe distortion of the desired polarization response. This result also shows that the proposed FF–DL method does not sacrifice the desired polarization response while improving interference suppression. The proposed loading mechanism mainly acts on the reliability of the joint covariance matrix and thus improves robustness without introducing obvious polarization-domain distortion.

Figure 8b further illustrates the joint influence of the input SNR and the number of snapshots on the proposed FF–DL method. The output SINR increases with the input SNR and also improves as more snapshots become available. A low–SINR region appears when the number of snapshots is extremely small, which is consistent with the severely underdetermined covariance-estimation condition discussed above. When the number of snapshots becomes sufficient for the enlarged joint spatial–polarization covariance matrix, the proposed method rapidly enters a stable high-SINR region. This three-dimensional result confirms that the proposed method is jointly affected by the signal power and the covariance-estimation reliability. The adaptive loading rule becomes most effective when the covariance estimate contains enough sample support but still requires regularization to suppress ill-conditioning.

Figure 8c shows the SINR gain of the proposed FF–DL method over the conventional SMI method. Except for the extremely underdetermined snapshot region, the proposed method generally provides a positive SINR gain over SMI. The gain becomes more stable when the number of snapshots increases, indicating that the proposed loading rule can effectively improve the reliability of the covariance inversion once a minimum level of sample support is available. The low-gain region at very small K is caused by the strong regularization imposed by the reliability-driven loading factor. In this case, the algorithm behaves conservatively because the joint covariance estimate is highly unreliable. This observation is consistent with the snapshot-dependent SINR result and highlights the importance of interpreting the proposed method under the high-dimensional joint spatial–polarization covariance setting.

Figure 8d presents the three-dimensional normalized response in the joint spatial–polarization domain. The response surface preserves the desired main response while forming pronounced null regions around the jammer directions. The nulls extend along the polarization dimension, which demonstrates that the proposed method exploits both spatial and polarization degrees of freedom for interference suppression. This result visually confirms the advantage of joint spatial–polarization processing. Compared with purely spatial interpretation, the three-dimensional response shows that the proposed method can suppress interference not only by angular discrimination but also by polarization-domain adaptation.

### 4.5. Example 4: Jammer Suppression and Numerical Stability

**Figure 9:**
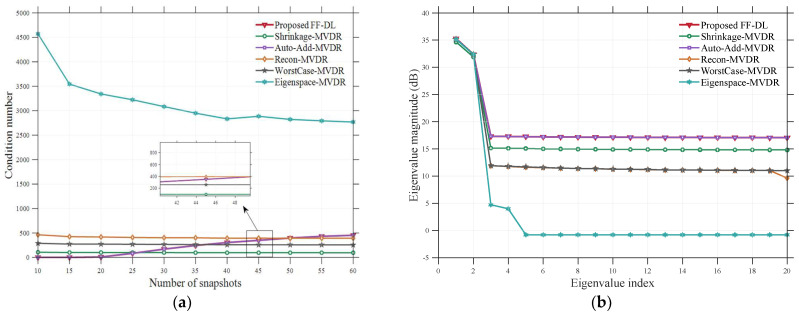
Jammer suppression and numerical stability. (**a**) Condition number versus number of snapshots; (**b**) eigenvalue spectra of covariance matrices.

The numerical stability of the inversion process is directly supported by the adaptive loading design. Figure 9a shows the covariance condition number versus the number of snapshots. The proposed method consistently maintains a smaller condition number than the benchmark methods, especially in the low-snapshot region. This directly supports the design logic of the adaptive loading term $\Pi_{c}$.

The eigenvalue spectra in Figure 9b further explain the SINR improvement. The proposed method suppresses excessively small eigenvalues that would otherwise amplify inversion noise, thereby producing a more regularized and stable covariance spectrum.

Figure 9a compares the condition numbers of the covariance matrices associated with different methods. Eigenspace–MVDR exhibits a much larger condition number than the other methods, indicating that the corresponding covariance representation remains highly ill-conditioned. In contrast, the proposed FF–DL method maintains the condition number at a moderately low level. Although its condition number is not the smallest among all methods, it achieves the best SINR performance, which indicates that excessive spectral compression is not necessarily beneficial. This result shows that the proposed method provides a balance between numerical stability and adaptive interference suppression capability. The reliability-driven loading factor improves the conditioning of the covariance matrix without overly suppressing the eigenstructure associated with the interference subspace.

Figure 9b shows the eigenvalue magnitude distribution. The benchmark methods exhibit different degrees of eigenvalue spreading and spectral compression. The proposed FF-DL method elevates the small eigenvalues via diagonal loading, thereby enhancing the numerical stability of the covariance matrix inversion. At the same time, it still preserves the dominant eigenvalue structure related to the jammer-plus-noise subspace. This eigenvalue behavior explains why the proposed method can achieve both robust covariance inversion and strong adaptive nulling. The loading factor is large enough to suppress ill-conditioning but not so excessive as to eliminate the useful covariance structure required by MVDR beamforming.

### 4.6. Example 5: Ablation Study

**Figure 10:**
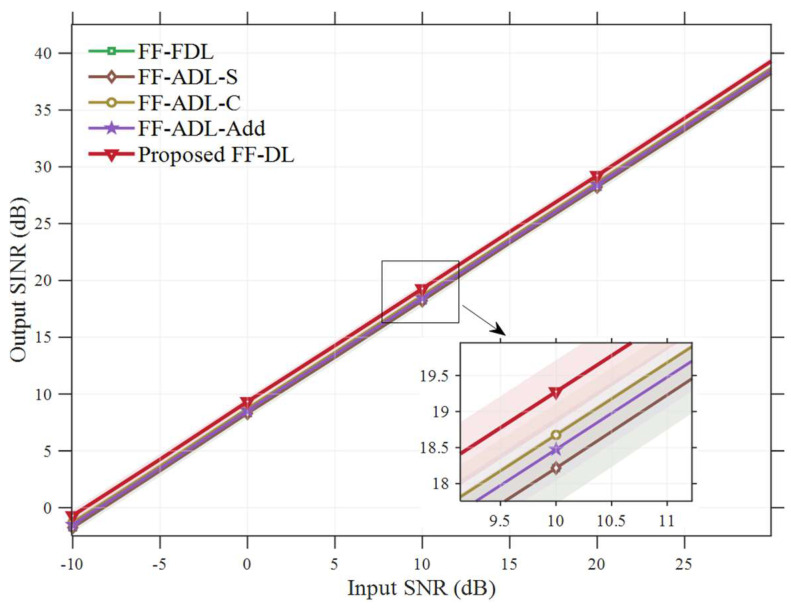
Ablation study versus input SNR.

Figure 10 presents the ablation results under different input SNRs. The proposed FF-DL method consistently achieves the highest output SINR among all ablated variants. Compared with FF–FDL, the improvement demonstrates the benefit of replacing a fixed loading level with an adaptive reliability-driven loading rule. Compared with FF–ADL–S and FF–ADL–C, the proposed method also performs better, indicating that snapshot deficiency and condition-number degradation provide complementary information for evaluating covariance reliability.

Moreover, the proposed method outperforms FF–ADL–Add, which uses an additive combination of the reliability terms. This result suggests that the adopted loading formulation can better reflect the joint effect of insufficient sample support and covariance ill-conditioning. Therefore, the full FF–DL design is more effective than using either reliability indicator alone or combining them in a simpler additive manner.

**Figure 11:**
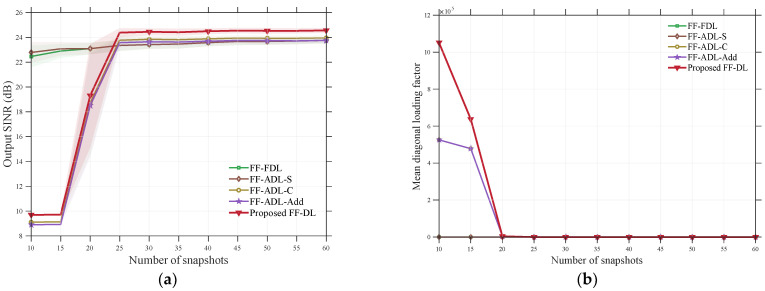
(**a**) Ablation study versus number of snapshots; (**b**) mean diagonal loading factor versus number of snapshots.

Figure 11a presents the ablation comparison as a function of the number of snapshots, while Figure 11b shows the corresponding mean diagonal loading factor. When the number of snapshots is extremely small, the proposed FF–DL method generates a very large loading factor because both snapshot deficiency and covariance ill-conditioning are severe. This strong regularization leads to a conservative beamforming solution and explains the low output SINR in the severely underdetermined region.

As the number of snapshots increases, the mean loading factor of the proposed method rapidly decreases. Once the covariance estimate obtains sufficient sample support, the proposed method recovers its adaptive capability and achieves the highest SINR among the ablated variants. This result verifies the intended behavior of the reliability-driven loading rule: strong regularization is applied when the covariance estimate is unreliable, whereas the loading level is reduced when the covariance estimate becomes more reliable.

Therefore, the proposed method is best understood as a reliability-aware regularization mechanism, rather than one that always maximizes SINR when the covariance estimate is severely underdetermined.

### 4.7. Example 6: Parameter Sensitivity Analysis

**Figure 12:**
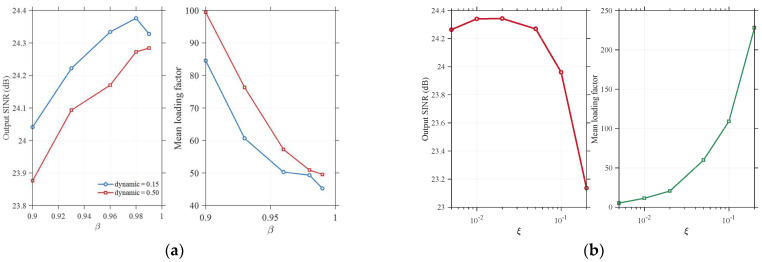
(**a**) Sensitivity to the forgetting factor; (**b**) sensitivity to the scaling parameter.

Figure 12a evaluates the influence of the forgetting factor β. As β increases, the covariance estimate uses a longer effective memory, and the required mean loading factor decreases accordingly. The output SINR generally improves with β and reaches a high level around β=0.96~0.98. This indicates that a moderately large forgetting factor can reduce covariance-estimation fluctuation while still preserving temporal tracking capability.

However, an excessively large β may slightly reduce the ability to track time-varying jammer statistics, which explains the mild SINR saturation or decrease near the largest tested value. In addition, the case with a smaller dynamic parameter gives a higher SINR and a lower loading factor than the case with a larger dynamic parameter. This suggests that overly aggressive dynamic loading may lead to unnecessary regularization and thus reduce the adaptive interference-suppression gain.

Figure 12b studies the sensitivity to the scaling parameter ξ. The output SINR remains relatively stable when ξ is within a small-to-moderate range. In this region, the loading factor is sufficient to improve covariance conditioning but does not dominate the MVDR optimization. When ξ becomes too large, the mean loading factor increases rapidly, and the output SINR decreases noticeably.

This result indicates that ξ controls the overall strength of the diagonal loading. A moderate value should be selected to balance numerical robustness and adaptive nulling capability. Excessive loading makes the beamformer less adaptive and therefore degrades the output SINR.

**Figure 13:**
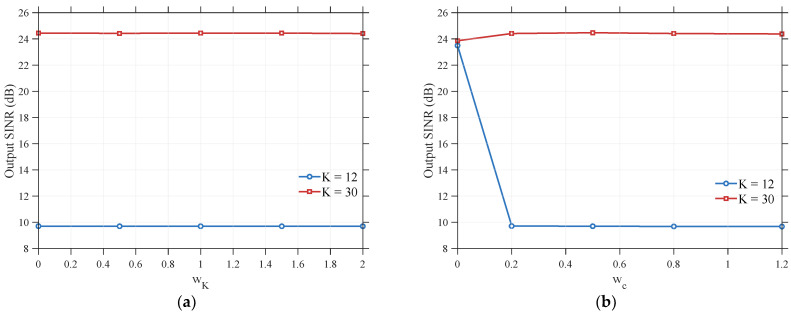
Sensitivity to weighting parameters. (**a**) Sensitivity to $w_{K}$; (**b**) sensitivity to $w_{c}$.

Figure 13 investigates the sensitivity to the weighting parameters $w_{K}$ and $w_{c}$. The output SINR is relatively insensitive to $w_{K}$ in both the moderate-snapshot case and the severely small-snapshot case. This indicates that the snapshot-deficiency weight primarily provides a consistent reliability adjustment and does not dominate the loading behavior within the tested range.

In contrast, $w_{c}$ has a more significant influence. For K=30, introducing the condition-number penalty either improves or maintains a high-output SINR, and the performance becomes stable once $w_{c}$ exceeds a small threshold. This confirms that condition-number information is useful for controlling covariance-inversion stability when sufficient sample support is available. However, for K=12, the covariance matrix is severely underdetermined, and the condition-number penalty triggers a very large loading factor. As a result, the beamformer becomes overly regularized and the output SINR decreases.

The two-dimensional sensitivity maps further confirm this observation. The mean loading factor increases mainly with $w_{c}$, while the influence of $w_{K}$ is relatively weak. The output SINR map shows that the performance transition is primarily governed by the condition-number weight. Therefore, $w_{c}$ should be selected carefully according to the minimum expected number of snapshots in practical applications.

The reference threshold $\kappa_{0}$ determines when the condition-number penalty becomes active. If $\kappa_{0}$ is too small, the condition-number penalty may be triggered too frequently, resulting in unnecessary diagonal loading. If $\kappa_{0}$ is too large, the penalty may remain inactive even when the covariance matrix is poorly conditioned. Therefore, $\kappa_{0}$ should be selected according to the typical condition-number range observed from calibration data or representative jammer-plus-noise secondary data. The floor term ε is only used to prevent the loading coefficient from vanishing in well-conditioned cases. Its influence is negligible as long as ε is much smaller than the data-dependent loading term.

It should also be noted that all simulation examples in this paper use the same parameter set specified in Section 4.1, without scenario-specific parameter adjustment. These examples include the input-SNR sweep, snapshot-number sweep, mismatch tests, ablation study, parameter-sensitivity analysis, high-INR analysis, and coherent-jammer scenario. This indicates that the selected parameters have reasonable robustness within the considered narrowband dual-polarized ULA simulation setting. However, for substantially different array geometries, array dimensions, jammer dynamics, or interference power distributions, moderate recalibration of μ, $\alpha_{c}$, and $\kappa_{0}$ may be required because the covariance eigenvalue distribution and condition-number behavior can change.

### 4.8. Example 7: High-INR Over-Regularization Analysis

**Figure 14:**
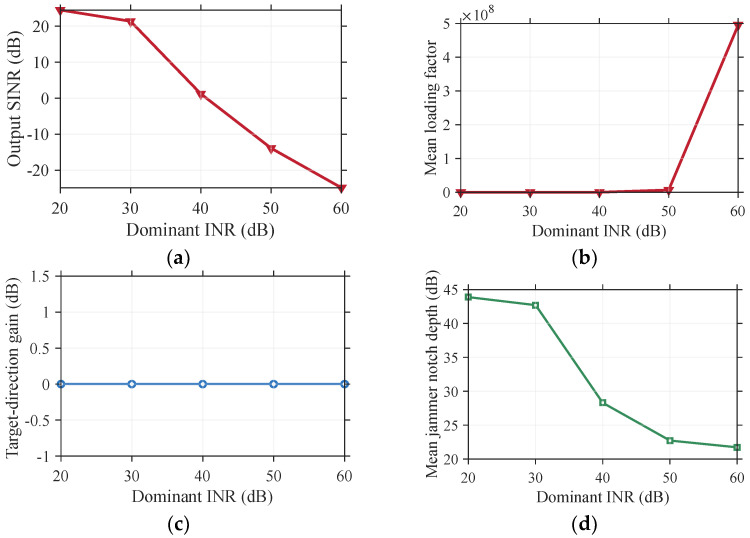
High-INR over-regularization behavior of the proposed FF-DL method. (**a**) Output SINR versus dominant INR; (**b**) Mean diagonal loading factor versus dominant INR; (**c**) Target-direction gain versus dominant INR; (**d**) Mean jammer notch depth versus dominant INR.

Figure 14 analyzes the behavior of the proposed FF-DL method under extremely high-INR conditions. Four performance indicators are considered: the output SINR, the mean adaptive loading factor, the target-direction gain, and the mean jammer notch depth.

Figure 14a shows the output SINR as a function of the dominant INR. When the dominant INR is 20 dB or 30 dB, the proposed method maintains a high-output SINR, indicating that the adaptive diagonal loading mechanism can regularize the covariance matrix and preserve interference suppression capability under moderately strong jamming conditions. When the dominant INR increases to 40 dB and above, however, the output SINR decreases significantly because the loading rule may become overly conservative and weaken adaptive null formation. This result reveals a practical performance boundary of the proposed reliability-dependent loading rule, rather than universal superiority under all jamming powers. It also motivates future work on upper-bounded or smoothed loading strategies to reduce over-regularization in extremely high-INR scenarios.

Figure 14b gives the corresponding mean loading factor. The loading factor remains moderate at relatively low INR levels, but increases rapidly when the INR becomes extremely large. This is because the loading coefficient is jointly determined by the covariance energy scale and the reliability penalties. Under very strong interference, both the covariance energy scale and the condition-number-related penalty may increase, resulting in a large diagonal loading factor. This confirms the reviewer’s concern that the adaptive loading coefficient can become large in high-INR scenarios.

Figure 14c shows the target-direction gain. The gain remains close to 0 dB over the whole INR range. This result indicates that, under the matched steering–vector condition, the response in the presumed target direction is preserved. Therefore, the high–INR degradation is not caused by direct attenuation of the desired steering direction.

Figure 14d shows the mean jammer notch depth. As the dominant INR increases, the notch depth decreases noticeably. This indicates that excessive diagonal loading weakens the adaptive use of the jammer covariance structure and reduces the null–forming capability of the beamformer. Therefore, the main risk of high-INR over-regularization is the degradation of adaptive jammer suppression, rather than the attenuation of the desired signal response.

Overall, Figure 14 clarifies the practical boundary of the proposed loading rule. The proposed method can work effectively under moderate and strong jamming conditions, but when the INR becomes extremely large, the loading factor may grow excessively and cause over-regularization. In practical implementations, an upper-bounded or smoothed loading strategy can be adopted to avoid overly conservative regularization under such extreme conditions.

### 4.9. Example 8: Performance Under Coherent Jammer Scenarios

**Figure 15:**
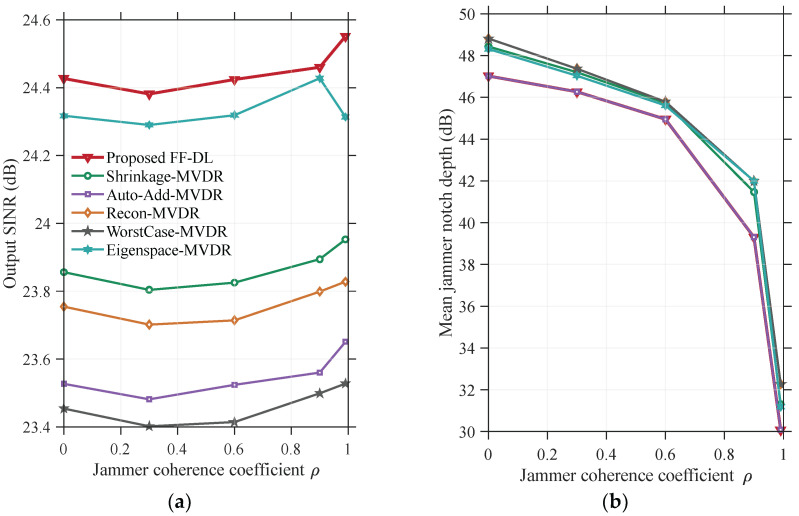
Performance under coherent jammer scenarios. (**a**) Output SINR versus jammer coherence coefficient ρ; (**b**) Mean jammer notch depth versus jammer coherence coefficient ρ.

Figure 15 evaluates the robustness of different methods under coherent jammer scenarios. The jammer coherence coefficient ρ is varied from 0 to 0.99. Here, ρ=0 corresponds to statistically independent jammers, while ρ close to 1 represents highly coherent jammers. The output SINR and the mean jammer notch depth are used as the evaluation metrics.

Figure 15a shows the output SINR versus the jammer coherence coefficient. The proposed FF-DL method achieves the highest output SINR over the whole range of ρ. Even when ρ approaches 0.99, the proposed method still maintains a stable SINR advantage over the benchmark methods. This result indicates that the proposed covariance regularization framework does not rely on the assumption that different jammers are statistically independent. When the jammers become highly coherent, the interference covariance matrix may become more rank-deficient or ill-conditioned. The proposed method can still provide a stable covariance inverse by combining forgetting-factor covariance tracking with reliability-driven diagonal loading.

Figure 15b shows the corresponding mean jammer notch depth. As ρ increases, the notch depth of all methods decreases, especially when the jammers become nearly coherent. This behavior is reasonable because coherent jammers change the rank structure of the interference covariance matrix. In this case, the beamformer mainly suppresses the coherent interference subspace rather than forming equally deep independent nulls for each jammer steering vector. Therefore, the decrease in individual jammer-direction notch depth does not necessarily mean that the overall interference suppression capability is completely lost.

Compared with the benchmark methods, the proposed FF-DL method maintains the best output SINR and competitive notch depth under partially coherent and highly coherent jammer conditions. These results demonstrate that the proposed method remains effective in coherent jammer scenarios, although the individual jammer-direction nulls may become shallower when the jammers are nearly coherent.

The simulation results demonstrate that the proposed FF-DL method achieves the smallest SINR loss relative to the optimal benchmark over a wide input-SNR range and provides strong robustness against moderate DOA and polarization mismatch. The beampattern and joint spatial–polarization response results confirm that the proposed method can form deep jammer nulls while preserving the desired response. The covariance condition number and eigenvalue analyses further show that the proposed method achieves a balance between numerical stability and adaptive interference suppression.

The ablation and parameter-sensitivity studies verify that the performance improvement comes from the joint use of forgetting-factor covariance tracking and reliability-driven diagonal loading. In particular, the comparison with Auto-DL-MVDR shows that the proposed method is not merely another automatic diagonal loading rule. Its advantage comes from making the loading coefficient responsive to both temporal covariance tracking and covariance reliability, including snapshot deficiency and condition-number degradation. It should also be noted that, under severely underdetermined snapshot conditions, the proposed rule may apply very strong loading and therefore behaves conservatively. When the number of snapshots becomes comparable to or larger than the joint covariance dimension, the proposed method rapidly recovers its adaptive capability and consistently outperforms the benchmark and ablated methods.

## 5. Conclusions

This paper proposed a robust polarization-domain adaptive anti-jamming method based on forgetting-factor covariance estimation and adaptive diagonal loading. The method improves MVDR beamforming from the covariance-estimation side. The forgetting-factor recursion enhances the tracking of time-varying jammer statistics, while the adaptive diagonal loading rule stabilizes covariance inversion by jointly accounting for sample deficiency and condition-number degradation. The main contribution of this work is to construct a covariance-reliability-driven regularization framework that combines temporal tracking and adaptive condition-number control for joint spatial–polarization anti-jamming.

Simulation results based on classical baselines and representative robust adaptive beamforming methods show that the proposed FF-DL method achieves competitive output SINR, stable covariance conditioning, and effective jammer suppression under the tested limited-snapshot and time-varying interference scenarios. Compared with covariance-reconstruction and uncertainty-set-based robust beamforming methods, the proposed method requires less prior information and avoids additional steering-vector reconstruction or uncertainty-set optimization. The proposed method is not intended to replace all existing robust beamforming methods, but rather provides a low-complexity covariance-reliability-driven alternative for polarization-domain anti-jamming.

The present validation is based on controlled simulations and should be regarded as first-stage algorithmic validation rather than complete hardware-level verification. Although the simulations include high-INR and coherent-jammer scenarios, several real-world effects, such as multipath propagation, antenna mutual coupling, array calibration errors, polarization-channel imbalance, hardware nonidealities, measured environmental clutter, and realistic propagation effects, are not explicitly modeled. Future work will consider measured-data validation, hardware-in-the-loop experiments, explicit steering-vector correction, knowledge-aided covariance regularization, and extensions to wideband or STAP scenarios.

## Figures and Tables

**Table 1:** Summary of the proposed robust polarization-domain adaptive anti-jamming algorithm.

Step	Operation
Step 1	Collect K jammer-plus-noise secondary snapshots and compute the forgetting-factor covariance estimate using (16)–(18).
Step 2	Compute μ, $\Pi_{K},$ and $\Pi_{c}$ from (19)–(21).
Step 3	Determine $\alpha_{prop}$ by (22) and form ${\hat{R}}_{prop}$ in (23).
Step 4	Obtain the robust MVDR weight $w_{prop}$ from (27).
Step 5	Evaluate output SINR, beampattern, polarization response, jammer notch depth, and conditioning indicators.

**Table 2:** Complexity comparison of the main methods.

Method	Computational Complexity
Shrinkage-MVDR	$O\left(KD^{2}+D^{3}\right)$
Auto-DL-MVDR	$O\left(KD^{2}+D^{3}\right)$
Recon-MVDR	$O\left(KD^{2}+GD^{2}+2D^{3}\right)$
Worst Case-MVDR	$O\left(KD^{2}+I_{\mathrm{wc}}D^{3}\right)$
Eigenspace-MVDR	$O\left(KD^{2}+2D^{3}\right)$
Proposed FF-DL	$O\left(KD^{2}+2D^{3}\right)$

**Table 3:** Main simulation parameters.

Parameter	Value
Number of spatial channels M	10
Polarization channels per element	2
Total dimension D	20
Inter-element spacing d	0.5 λ
Wavelength normalized λ	1
Target DOA $\theta_{s}$	10°
Target polarization angle $\gamma_{s}$	25°
Target phase difference $\eta_{s}$	0°
Jammer DOAs	−30°, 40°
Jammer polarization angles	70°, 35°
Jammer phase differences	60°, −80°
Noise variance $\sigma_{n}^{2}$	1
Nominal jammer INR	24 dB, 21 dB
SNR sweep	−10:5:30 dB
Snapshot sweep K	10:5:60
Monte Carlo trials	180

## Data Availability

The data presented in this study are available on request from the corresponding author.
